# FISH-aimed karyotype analysis in *Aconitum* subgen. *Aconitum* reveals excessive rDNA sites in tetraploid taxa

**DOI:** 10.1007/s00709-018-1238-9

**Published:** 2018-03-14

**Authors:** Andrzej J. Joachimiak, Robert Hasterok, Elwira Sliwinska, Krystyna Musiał, Aleksandra Grabowska-Joachimiak

**Affiliations:** 1 Department of Plant Cytology and Embryology, Institute of Botany, Jagiellonian University, Gronostajowa 9, PL-30-387 Kraków, Poland; 2 Department of Plant Anatomy and Cytology, University of Silesia in Katowice, Jagiellonska 28, 40-032 Katowice, Poland; 3 Laboratory of Molecular Biology and Cytometry, Department of Plant Genetics and Biotechnology, University of Technology and Life Sciences in Bydgoszcz, Kaliskiego 7, 85-789 Bydgoszcz, Poland; 4 Department of Plant Breeding and Seed Science, University of Agriculture in Kraków, Łobzowska 24, 31-140 Kraków, Poland

**Keywords:** *Aconitum*, Chromosomes, FISH, rDNA, Nuclear DNA amount, Diploidization

## Abstract

The location of 5S and 35S rDNA sequences in chromosomes of four *Aconitum* subsp. *Aconitum* species was analyzed after fluorescence in situ hybridization (FISH). Both in diploids (2*n* = 2*x* = 16; *Aconitum variegatum*, *A. degenii*) and tetraploids (2n = 4× = 32; *A. firmum*, *A. plicatum*), rDNA repeats were localized exclusively on the shorter arms of chromosomes, in subterminal or pericentromeric sites. All analyzed species showed similar basal genome size (Cx = 5.31–5.71 pg). The most striking features of tetraploid karyotypes were the conservation of diploid rDNA loci and emergence of many additional 5S rDNA clusters. Chromosomal distribution of excessive ribosomal sites suggests their role in the secondary diploidization of tetraploid karyotypes.

## Introduction

Almost all *Aconitum* species studied so far have an interesting bimodal karyotype (*x* = 8) with two large (metacentric and submetacentric) and six small (usually submetacentric) chromosomes (Yuan and Yang 2006, Hong et al. 2017). Most taxa are diploid (2*n* = 16) or tetraploid (2*n* = 32), but triploid, pentaploid, hexaploid, and octoploid plants have been also detected (Simon et al. 2001). Only diploid *A. fletcherianum* (possessing two large metacentric chromosome pairs and *x* = 6) has a different genome construction (Hong et al. 2016). It seems puzzling that during 30 million years of evolution of so widely distributed and species-rich genus (Jabbour and Renner 2012), its karyotype uniformity has been preserved.

*Aconitum* karyotypes can show marked differences in the amount of heterochromatin, especially at sites localized within bands associated with nuclear organizers. From three diploid taxa belonging to the subgenus *Aconitum*, *A. degenii* and *A. lasiocarpum* showed a heterochromatin-poor karyotype with relatively stable C-banding patterns, while *A. variegatum* generally showed a higher amount of heterochromatin (Joachimiak et al. 1999). Two high-mountain tetraploid species belonging to this subgenus, Western Carpathian *A. firmum* and Sudetic *A. plicatum*, also contained different amounts of heterochromatin (Mitka et al. 2007). This suggests that *Aconitum* karyotypes, although stable at the level of chromosome morphology, are differentiated at the intra-chromosomal level. The existence of such differences has much potential use in taxonomical and phytogeographical studies of *Aconitum*, especially because of the general lack of reproducible, species-specific molecular markers within this genus. It has been shown that many well-defined or geographically disjunct *Aconitum* taxa possess identical or nearly identical ITS (internal transcribed spacer) and cpDNA sequences (Kita et al. 1995; Kita and Ito 2000; Utelli et al. 2000; Luo et al. 2005; Mitka et al. 2016). Although some arbitrarily amplified sequences (RAPD, ISSR) seem to be useful for the estimation of molecular diversity of target *Aconitum* genomes (Fico et al. 2003; Zhang et al. 2005; Mitka et al. 2007), low reproducibility and the generation of dominant, non-locus-specific markers limit the broader applicability of these methods in phylogenetic studies.

Almost all the karyotypic data on *Aconitum* are restricted to chromosome number and morphology, DAPI/CMA fluorochrome staining, and C-banding patterns (Okada 1991; Joachimiak et al. 1999; Simon et al. 2001; Ilnicki 2005; Mitka et al. 2007). There is no information on the localization of marker sequences in chromosomes, molecular composition of heterochromatic bands, and nuclear genome size of this genus. All this information could be useful in the identification of chromosomes, evaluation of intra- and inter-specific diversity, and acquiring knowledge on the origin and evolution of polyploid species. In the present study, we analyzed karyotypes of two diploid (*A. variegatum* and *A. degeni*) and two tetraploid (*A. firmum* and *A. plicatum*) species of *Aconitum* belonging to the subgen. *Aconitum* by fluorescence in situ hybridization with 35S rDNA and 5S rDNA probes. Chromosomal localization of rDNA sequences, along with information on the C-banding patterns (Joachimiak et al. 1999; Ilnicki 2005; Mitka et al. 2007), was used for more precise characterization of karyotype in these species. Additionally, the genome size of analyzed species was established by flow cytometry.

## Materials and methods

### Plant material and chromosome preparation

The materials for the study were specimens of *Aconitum variegatum* L. and *A. degenii* Gáyer (2n = 2× = 16), *A. firmum* Rhb., and *A. plicatum* Köhler ex Rchb. (2n = 4× = 32) belonging to the subgenus *Aconitum*, collected from natural sites in Carpathians and Sudetes and then cultivated in the Botanical Garden of the Jagiellonian University in Kraków. All of the analyzed species are rare and protected in Poland. Three of them (*A. degenii*, *A. firmum*, and *A. plicatum*) are listed in the Polish Red Data Book of Plants (2001) and classified as vulnerable species. *A. variegatum* is a Central European endemic representing the mountain element in the lowlands, and *A. degenii* is an endemic of eastern and southern Carpathians. From the two high-mountain tetraploid taxa, *A. firmum* is a western Carpathian endemic, and *A. plicatum* is a central European endemic known in Poland from the Sudetes. Three plants of Polish origin from each species were analyzed by FISH. Cytometric DNA estimations were performed not only on these plants but also on material collected in other Carpathian sites (Table 1).

**Table 1:** The origin of investigated plants. Taxonomical nomenclature after Mitka (2003)

Species	Origin	Number of analyzed plants	
		DNA amount	FISH
*A. variegatum*	Gorce Mts	1	1
	Beskid Niski Mts	3	1
	Dąbrowa Górnicza	5	–
	Krzymosze near Siedlce	1	1
	Transsylvania	2	–
*A. degenii*	Bihor Mts	1	–
	Gutii Mts	1	–
	Czarnohora Mts	1	–
	Bieszczady Mts	3	3
*A. firmum*	Rodna Mts	1	–
	Tatra Mts	3	3
*A. plicatum*	Sudeten Mts	3	3

Plants were taken to the laboratory and kept in pots, and then tips of roots were cut from them for cytological analysis. The material was pretreated with saturated aqueous solution of α-bromonaphtalene overnight at 4 °C and fixed in absolute ethanol/glacial acetic acid. After fixation, root tips were macerated with a 1% mixture of pectinase and cellulase for 10 min at 37 °C. Preparations were made by squashing in a drop of 45% acetic acid. Cover slips were then flicked off after freezing in dry ice, air-dried, and kept at − 24 °C until used.

### DNA probe labeling and FISH

The procedure followed the protocol described in detail by Wolny and Hasterok (2009) with minor modifications. In brief, the following DNA probes were used: (i) a 2.3 kb *ClaI* subclone representing the fragment of a 25S rDNA genic region of *Arabidopsis thaliana* (Unfried and Gruendler 1990) was labeled by nick translation with digoxigenin-11-dUTP (Roche, Indianapolis, IN, USA) and used to detect the 35S rDNA loci containing the genes encoding for 18S-5.8S-25S rRNA. (ii) The pTa794 clone containing a 410 bp fragment of 5S rDNA unit isolated from wheat (Gerlach and Dyer 1980) was labeled by PCR with tetramethyl-rhodamime-5-dUTP (Roche) and used to visualize 5S rDNA loci. Primer sequences and conditions for the reactions were as described by Hasterok et al. (2002).

General conditions of the FISH procedure were as follows: slides were incubated with DNase-free RNase (100 μg/ml) in 2× SSC for 1 h at 37 °C, then washed in three changes of 2× saline sodium citrate (SSC) buffer for 15 min, post-fixed in 1% formaldehyde in PBS buffer followed by washes in 2× SSC for 15 min, dehydrated in an ethanol series (70, 90, and 100%) and air-dried. The DNA probes were mixed to a final concentration of 2.5–3.5 ng/μl of hybridization mixture along with 50% deionized formamide, 20% dextran sulfate, 2× SSC, 0.5% SDS, and salmon sperm blocking DNA in 500–100× excess of labeled probes. The hybridization mixture was pre-denatured (75 °C for 10 min), applied to the chromosome preparations, and denatured together at 75 °C for 5 min in an Omnislide in situ hybridization system (Hybaid, Basingstoke, UK) and then incubated overnight at 37 °C in a humid chamber to allow renaturation. After hybridization, slides were washed for 10 min in 15% deionized formamide in 0.1× SSC at 42 °C, which is equivalent to 82% stringency, followed by several washes in 2× SSC. Digoxigenated probe was immunodetected according to standard protocols by antidigoxigenin antibodies conjugated with fluorescein isothiocyanate (FITC; Roche). After final dehydration, preparations were mounted and counter-stained in Vectashield (Vector Laboratories, Burlingame, CA, USA) antifade buffer containing 2.5 μg/ml 4′-6-diamidino-2-phenylindole (DAPI; Serva). Images were taken using a Leica DMRB epifluorescent microscope equipped with a *CoolSnap* (Photometrics) monochromatic CCD camera and then processed uniformly using Picture Publisher (Micrographx) software. For visualization of very tiny FISH signals, we used the option “find edges” of the ImageJ program (http://rsb.info.nih.gov/ij/).

The karyograms with marked FISH-detected rDNA segments were constructed on the basis of the generalized karyotype (Table 2) of four *Aconitum* species. It was obtained from the measurements of 40 DAPI-stained metaphases (10 per each species). For the heteromorphic pairs/groups of chromosomes, additive chromatin segments (0.2–0.3 μm long) were taken into account.

**Table 2:** Measurements of *Aconitum* chromosomes (in μm and in % of genome length). *ChT* chromosome type, *L* longer arm, *S* shorter arm, *T* total length, *Ar* arm ratio (L/S), *C* centromere localization

ChT	L		S		T		Ar	C
	μm	%	μm	%	μm	%		
1	5.20	12.92	4.18	10.39	9.38	23.30	1.24	m
2	5.72	14.21	2.65	6.59	8.37	20.80	2.16	sm
3	2.60	6.46	1.95	4.84	4.55	11.30	1.33	m
4	2.83	7.03	1.31	3.26	4.14	10.29	2.16	sm
5	2.95	7.33	1.06	2.63	4.01	9.96	2.78	sm
6	2.80	6.96	1.04	2.58	3.84	9.54	2.69	sm
7	2.48	6.16	1.03	2.56	3.51	8.72	2.41	sm
8	1.77	4.40	0.68	1.69	2.45	6.09	2.60	sm

### Nuclear DNA content measurements

For the flow cytometric estimation of nuclear DNA content, *Pisum sativum* cv. Set (2C = 9.11 pg; Sliwinska et al. 2005) was used as an internal standard. Young leaves derived from adult plants were used as material for the study. Plant tissues of *Aconitum* and *P. sativum* were chopped simultaneously with a sharp razor blade in a plastic Petri dish with 1 ml nucleus-isolation buffer LB01 (Doležel et al. 1989), supplemented with propidium iodide (50 μg/ml) and ribonuclease A (50 μg/ml). After chopping, the suspension was passed through a 50-μm mesh nylon filter. For each sample, 6000–10,000 nuclei were analyzed directly after preparation using a Partec CCA flow cytometer (Münster, Germany). Analyses were replicated three times for each specimen. Histograms were analyzed using a DPAC v.2.2 computer program. Absolute nuclear DNA content was calculated using the linear relationship between the ratios of the 2C peak positions *Aconitum*/*Pisum*, on the histogram of fluorescence intensities (Galbraith et al. 1998).

## Results

### Diploids

**Fig. 1:**
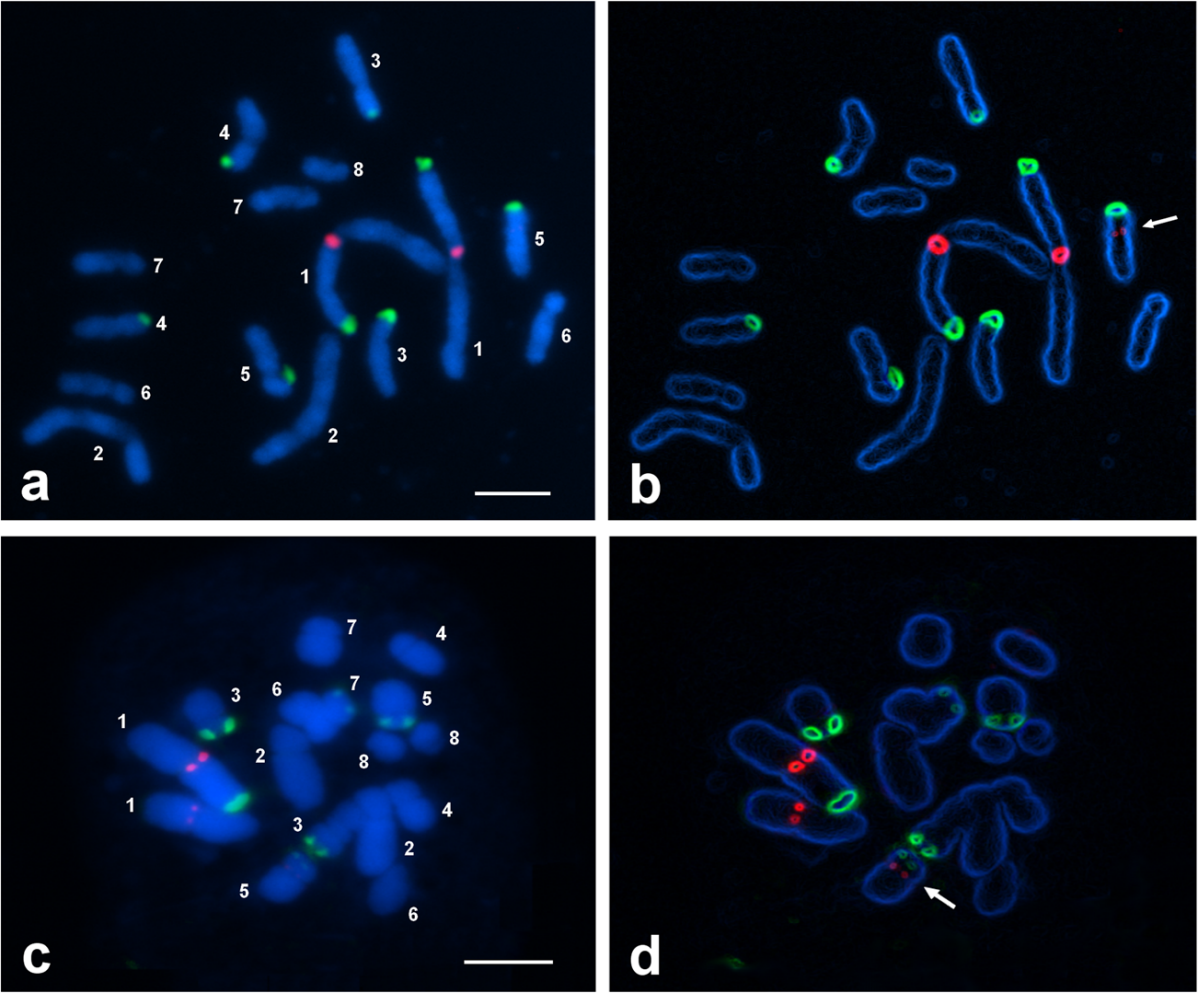
Fluorescence in situ hybridization of 35S rDNA (green) and 5S rDNA (red) probes to the somatic metaphase chromosomes of **a**, **b** *A. variegatum* and **c**, **d** *A. degenii*. Images **b** and **d** were processed with the use of the option “find edges” of the ImageJ program. The chromosomes were counter-stained with DAPI (blue). Arrows indicate small 5S rDNA site in chromosome 5, detected after image processing. Bar 5 μm

**Fig. 2:**
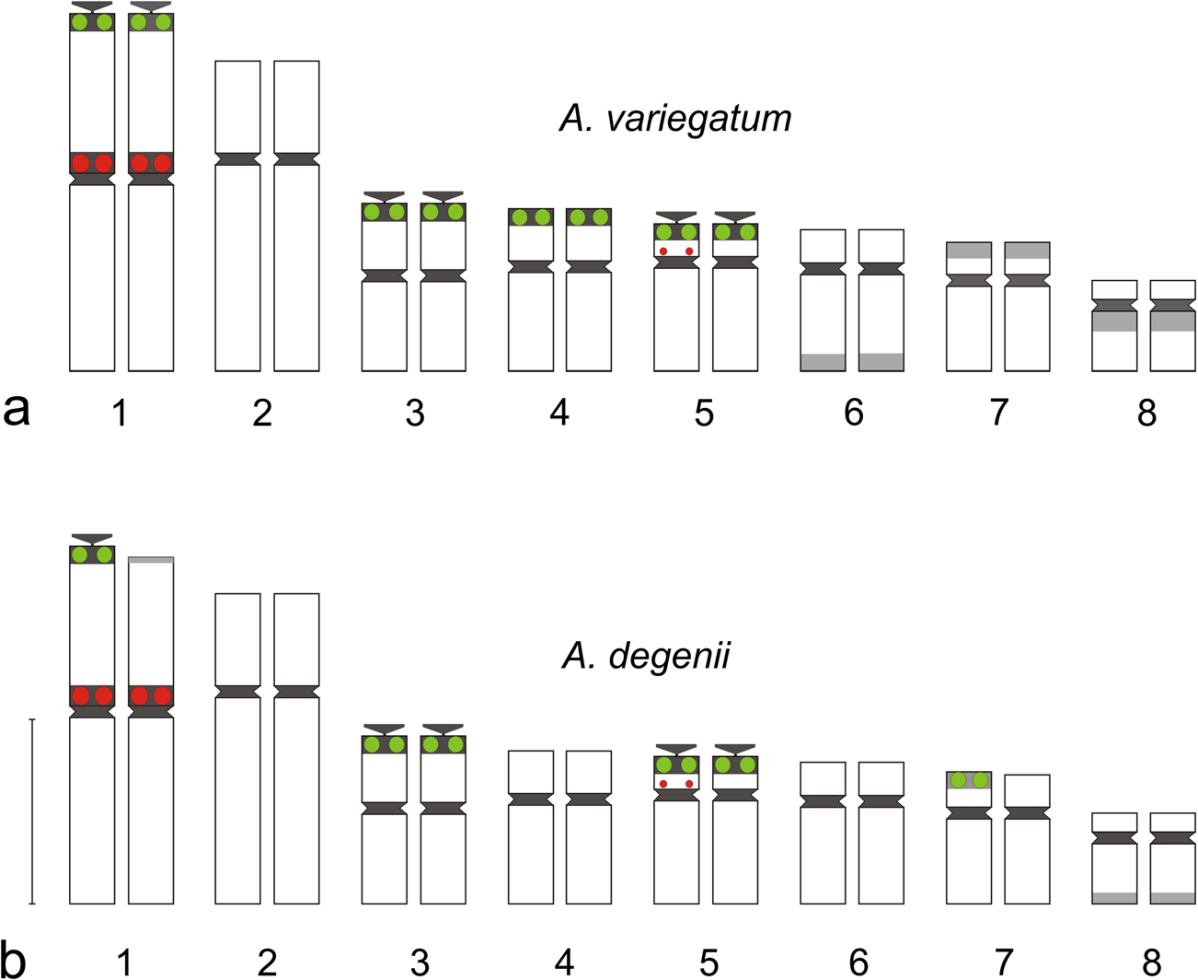
Karyotype structure of *A. variegatum* (**a**) and *A. degenii* (**b**). Green—35S rDNA, red—5S rDNA, black—fixed heterochromatin segments, gray—polymorphic heterochromatin segments (heterochromatin classification according to Joachimiak et al. 1997). The position of satellites and heterochromatin was taken from Joachimiak et al. (1999), Ilnicki (2005), and Mitka et al. (2007). Bar 5 μm

The two diploid species showed similar distribution of rDNA sites within the karyotype. All detected 35S rDNA sequences were localized terminally and 5S rDNA sequences pericentromerically. There were eight 35S rDNA signals within the chromosome complement of *A. variegatum*, localized on the shorter arms of four-chromosome pairs (1, 3, 4, and 5) (Figs. 1a, b and 2a). In *A. degenii*, only six 35S rDNA sites were detected. Four of them were localized on chromosomes 3 and 5, one on chromosome 1 and one on chromosome 7 (Figs. 1c, d and 2b). 35S rDNA clusters on chromosomes 1, 3, and 5 seem to be the major nucleolar-organizing regions (NORs) in the analyzed plants. A specific feature of the *A. variegatum* karyotype was the occurrence of two distinct 35S rDNA signals in the non-satellited chromosome pair 4 (Fig. 2a)*.*

Both *A. variegatum* and *A. degenii* showed two large 5S rDNA sites localized within the pericentromeric heterochromatin of the shorter arms of the longest chromosomes (pair 1) (Figs. 1 and 2). Additionally, chromosome 5 may harbor a very tiny 5S rDNA signal near centromere, practically undetectable without special image processing. Only one chromosome 5 with such signal was detected in both species, indicating that 5S rDNA repeats were absent or at a copy number below detection level on the homolog.

The nuclear 2C DNA amount was estimated at 11.43 pg in *A. variegatum* and 11.23 pg in *A. degenii*, so the basal genome size is nearly identical in both specie (5.71 and 5.61 pg, respectively) (Table 3).

**Table 3:** Nuclear DNA content in *Aconitum* species

Species	Ploidy	DNA amount (pg ± SD)	
		2C	1Cx
*A. variegatum*	2×	11.43 ± 0.11	5.72
*A. degenii*	2×	11.23 ± 0.14	5.62
*A. firmum*	4×	21.62 ± 0.14	5.41
*A. plicatum*	4×	21.24 ± 0.25	5.31

### Tetraploids

**Fig. 3:**
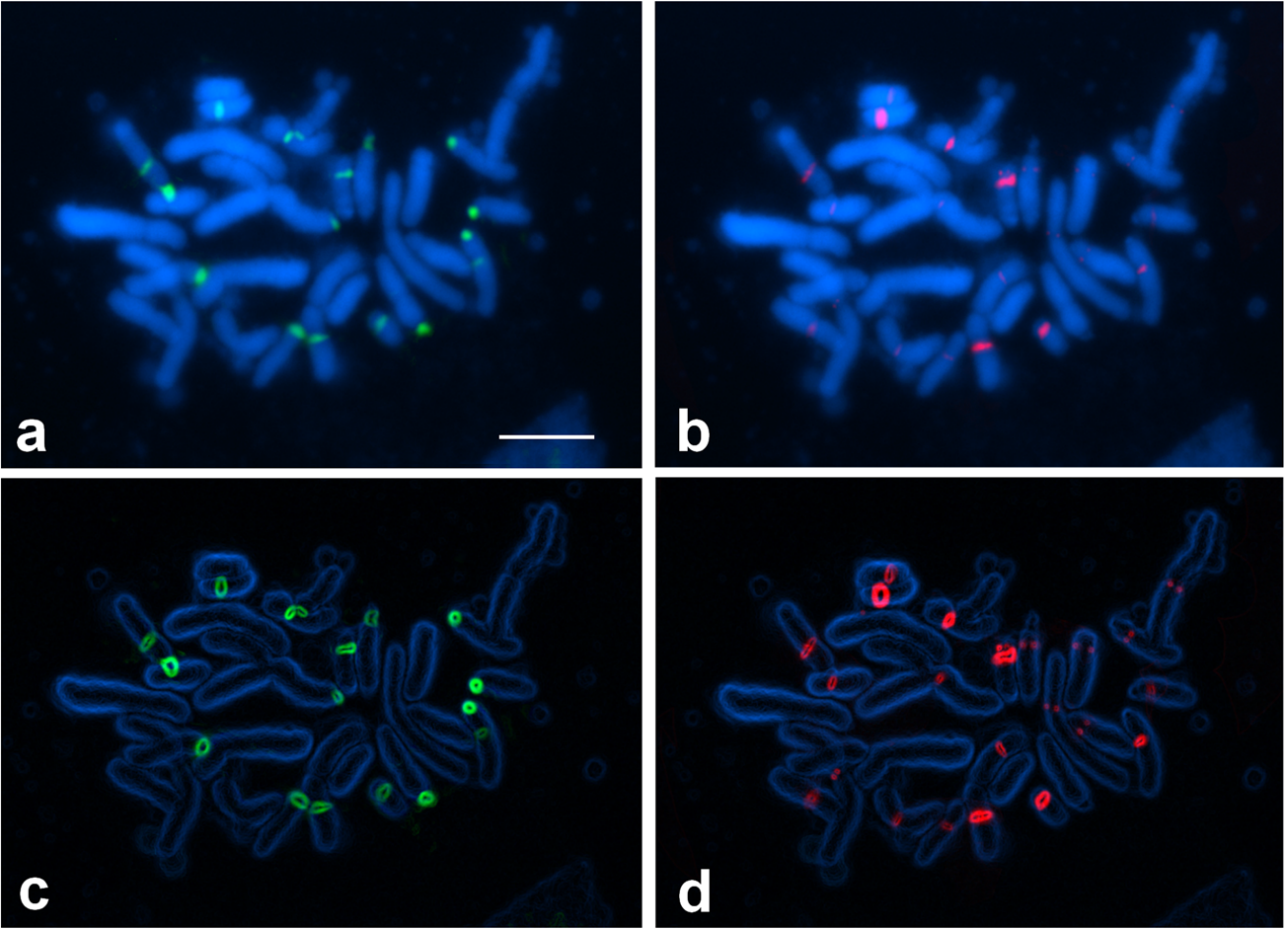
Fluorescence in situ hybridization of 35S rDNA (green) and 5S rDNA (red) probes to the somatic metaphase chromosomes of *A. firmum*. The chromosomes were counter-stained with DAPI (blue). **a**, **b** Before processing and **c**, **d** after processing in the ImageJ software. Bar 5 μm

**Fig. 4:**
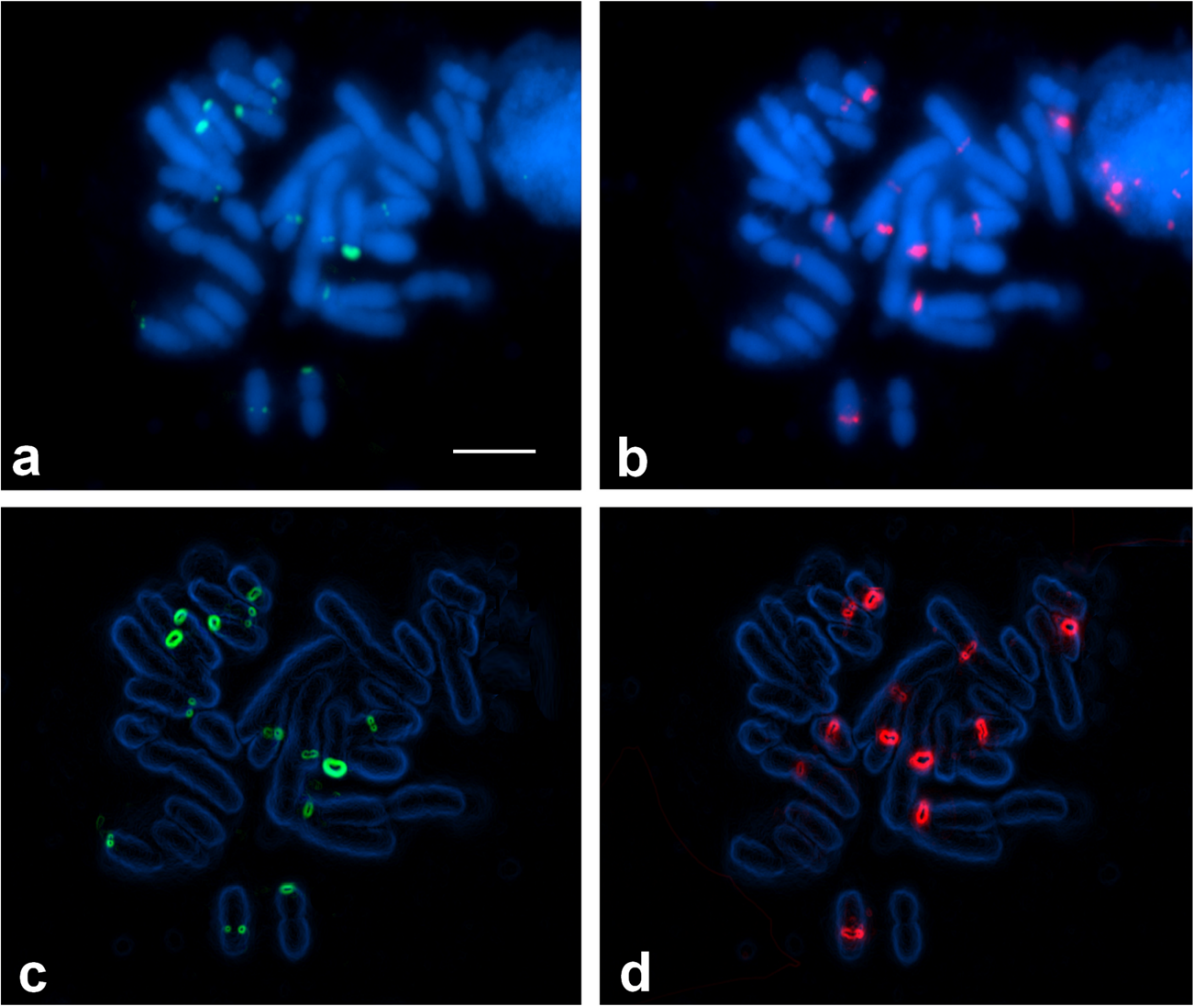
Fluorescence in situ hybridization of 35S rDNA (green) and 5S rDNA (red) probes to the somatic metaphase chromosomes of *A. plicatum*. The chromosomes were counter-stained with DAPI (blue). **a**, **b** Before processing and **c**, **d** after processing in the ImageJ software. Bar 5 μm

**Fig. 5:**
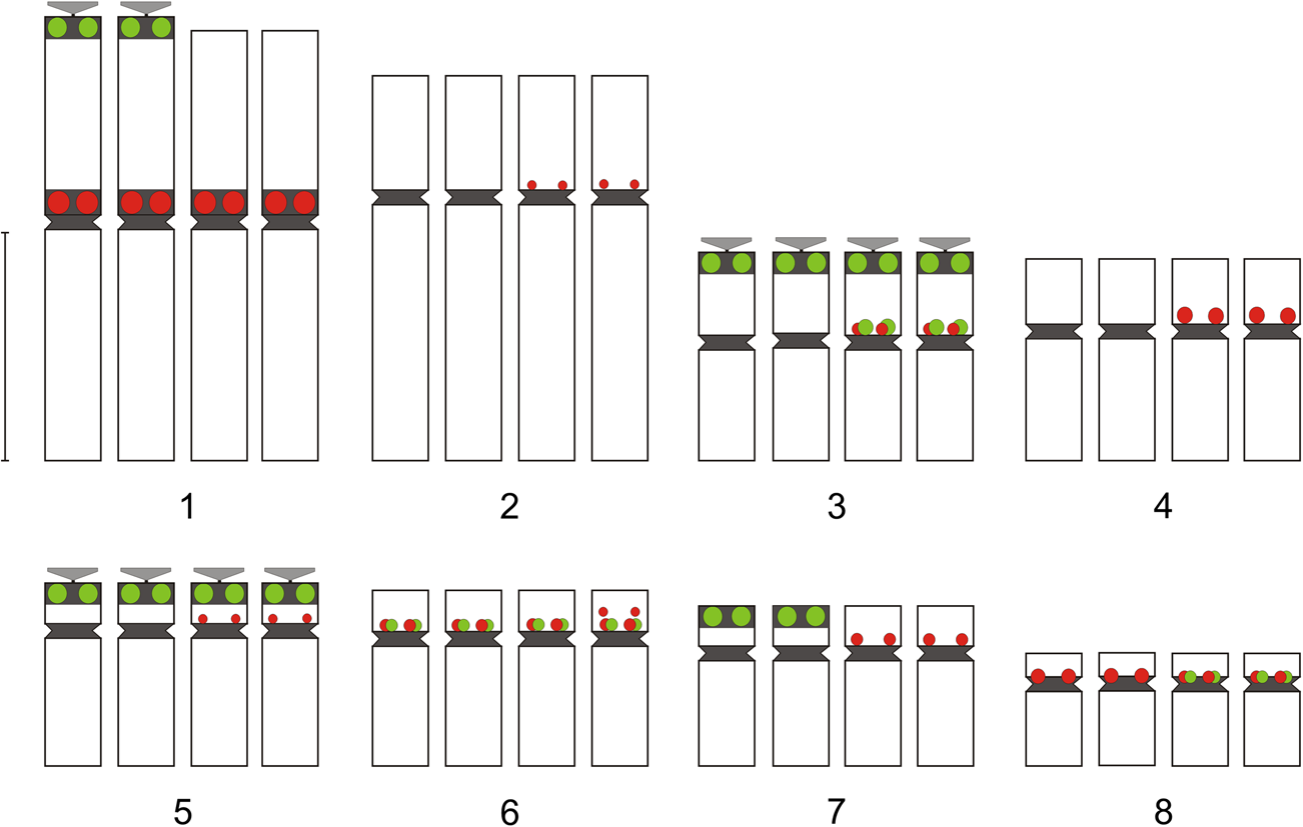
Localization of 35S rDNA (green) and 5S rDNA (red) in the chromosomes of *A. firmum*. Only fixed heterochromatin segments were considered (black). The position of satellites and heterochromatin was taken from Joachimiak et al. (1999), Ilnicki (2005), and Mitka et al. (2007). Bar 5 μm

**Fig. 6:**
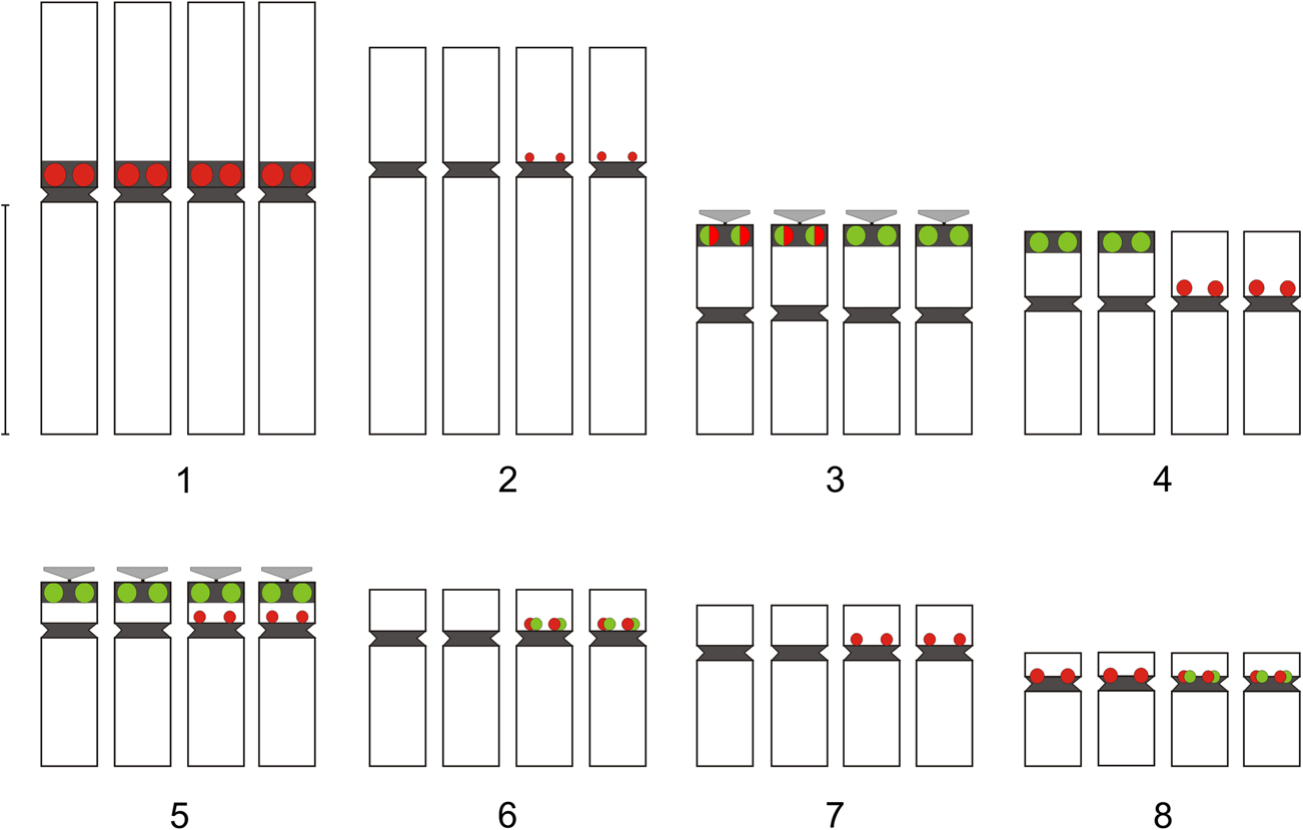
Localization of 35S rDNA (green) and 5S rDNA (red) in the chromosomes of *A. plicatum*. Only centric and rDNA-associated heterochromatin is considered (black). The position of satellites and heterochromatin was taken from Joachimiak et al. (1999) and Mitka et al. (2007). Bar 5 μm

The two tetraploid species analyzed here showed a high number of 35S and 5S rDNA loci (Figs. 3 and 4). However, some rDNA loci were small and thus not detectable in all preparations. The distribution of 5S rDNA signals was similar to that observed in diploids: almost all detected sequences were located pericentromerically. 35S rDNA clusters were located not only terminally but also pericentromerically. In all instances, pericentromeric clusters of 35S rDNA colocalized with 5S rDNA (Figs. 5 and 6).

The number of detected 35S rDNA signals was up to 20 in *A. firmum* and up to 14 in *A. plicatum*. The most characteristic feature of Carpathian *A. firmum* was the heterozygosity of the first and third four-chromosome group in respect of the 35S rDNA distribution (Fig. 5). The karyotype of this species showed major 35S rDNA clusters on the ends of two chromosomes of type 1 and in the pericentromeres of two satellited chromosomes of type 3. The same chromosome sites in Sudetic *A. plicatum* were invariably lacking 35S rDNA signals (Fig. 6).

When compared to the two diploid species, the number of 35S rDNA loci in *A. plicatum* (14) did not show clear deviation from additivity. *A. firmum* showed, however, more 35S rDNA signals (20) than expected from the karyotypes of the two analyzed Carpathian diploids (6–8).

The most striking feature of the tetraploid karyotypes is the presence of a huge number of 5S rDNA clusters, located in chromosomes belonging to all morphological groups. Both analyzed tetraploids showed a similar pericentromeric distribution of these repeats. The number of detected 5S rDNA signals was up to 23 in *A. firmum* and up to 20 in *A. plicatum*. Many of these signals were small and hardly detectable. The most prominent were located in the pericentromeric region of the shorter arms of chromosomes 1 (4 signals), 4 (2 signals), and 8 (2 signals).

The nuclear DNA content estimated for *A. firmum* was 2C = 21.62 pg and 2C = 21.24 pg for *A. degenii*, so the basal genome size is nearly identical in both species (Cx = 5.40 pg and Cx = 5.31 pg, respectively) (Table 3).

## Discussion

In this paper, we presented for the first time a thorough FISH analysis of the rDNA sites in *Aconitum*. Together with the previously performed chromosome studies, it provides interesting data on the karyotype structure and evolution in this species-rich and widely spread genus.

The majority of terminal 35S rDNA sites detected in analyzed species by FISH were frequently observed as small satellites in conventionally stained preparations (Joachimiak et al. 1999; Ilnicki 2005). A similar localization was reported for chromomycin A_3_-positive and DAPI-negative (CMA+/DAPI−) chromatin segments in the Japanese diploid species *A. sanyoense* (Okada 1991). In the majority of plant species, CMA+/DAPI− staining is characteristic for NOR-associated chromatin (Guerra 2000), so it can be deduced that the distribution of major NOR loci was the same in all three diploid species. According to Roa and Guerra (2012), the stability of rDNA sites is common phenomenon within taxa characterized by conserved chromosome morphology.

The terminal 35S rDNA sites were also observed on the non-satellited chromosome pair 4 in *A. variegatum*. The presence of 35S rDNA repeats has frequently been reported in regions where secondary constrictions have not been located (Guerra 2000, Roa and Guerra 2012). Most likely, they represent inactive, epigenetically silenced rDNA sites. Such silencing of rDNA clusters located out of NOR chromosomes was demonstrated in *Rumex acetosa* where 35S rDNA on the non-satellited chromosome pair was highly methylated (Lengerova and Vyskot 2001).

All terminally located 35S rDNA sites detected here were previously described as C-band positive (Joachimiak et al. 1999; Ilnicki 2005). They constituted (together with the major 5S rDNA cluster localized near centromere of chromosome 1) the major heterochromatic loci in C-banded *Aconitum* genomes. Although different plants belonging to the analyzed taxa showed more C-band-positive segments, only some of these segments were conserved (i.e., observed in all or nearly all chromosomes of a given type). The most stable were those located at the centromeres and NORs. Interestingly, polymorphic heterochromatin in *Aconitum* does not generally contain rDNA, and all minor rDNA sites in diploids as well as minor and extra rDNA sites in tetraploids were not heterochromatinized.

*A. degenii* chromosome pair 1, detected here as heterozygous in respect of the presence of 35S rDNA on the end of the shorter arm, was also heterozygous in respect of the amount of heterochromatin at this position (Fig. 2b). Heterochromatin difference between homologs was stable and observed in many plants collected from very distant sites in Poland, the Ukraine, and Romania (Joachimiak et al. 1999; Ilnicki 2005). The same phenomenon was observed in *A. lasiocarpum*, a close relative of *A. degenii* (Joachimiak et al. 1999). The reason why plants with homomorphic pair 1 were not observed in analyzed material remains unknown. The absence of 35S rDNA in one chromosome of type 1 and the presence of this sequence in chromosome 7 in *A. degenii* suggested a 1:7 translocation of nucleolar-organizing region (Fig. 2b). The analysis of meiosis in plants belonging to the sect. *Lycoctonum* performed many years ago by Afify (1933) showed that chromosome translocations are not uncommon in *Aconitum* taxa. The structural heterozygosity of the first chromosome group was also observed in tetraploid *A. firmum*, both in FISH and C-banding studies.

A high number of rDNA sites observed in the analyzed tetraploids is a very interesting phenomenon because the general trend in chromosome evolution seems rather to be partial reduction of rDNA loci in polyploid species (Maluszynska and Heslop-Harrison 1993; Volkov et al. 1999; Krishnan et al. 2001; Mishima et al. 2002; Weiss-Schneeweiss et al. 2007; Roa and Guerra 2012). The important steps in the lowering of such loci were the inactivation of one of the parental rDNA and a gradual decrease in the number of rDNA repeats. Such changes, resulting in asymmetry or loss of rDNA clusters, were suggested in *Brassica* (Hasterok et al. 2006), *Trifolium* (Ansari et al. 2008), and *Nicotiana* (Kovarik et al. 2008). Variation in the number and position of rDNA repeats could result from the changes of chromosomes such as ectopic (interlocus) recombination, inversions and translocations, or transposition (Gernand et al. 2007). It has been suggested that the first two events are influenced by chromosomal location: terminal localization allows frequent rearrangement to occur without disrupting other gene linkages (Hanson et al. 1996). This is supported by observations in *Gossypium* and *Nicotiana* where sub-telomeric 35S rDNA loci show interlocus homogenization, while interstitial 5S rDNA loci do not (Cronn et al. 1996; Fulnecek et al. 2002). The lack of detectable changes in chromosome morphology and uniform pericentromeric location of 5S rDNA in tetraploid *Aconitum* plants suggest rather the involvement of transposition and further amplification of transposed rDNA sequences. The ability of rDNA sequences to transpose was reported by Schubert and Wobus (1985), Adams et al. (2000), Raskina et al. (2004), Datson and Murray (2006). It was supposed that mobility of repeated sequences may result from activation of dormant transposons by different stresses, including polyploidy and in vitro culture (Capy et al. 2000; Liu and Wendel, 2002; Gernand et al. 2007; and references cited therein). They can also be incorporated into transposable elements, as it was revealed specifically for 5S rDNA by Kalendar et al. (2008) or directly amplified as a satellite (Martins et al. 2006). The role of massive amplification of rDNA loci, observed only in some plant species, remains unknown. Most probably, it enhances the differences between similarly shaped chromosome pairs, as in *Tulipa* (Mizuochi et al. 2007), or reinforces the chromosome-arm homology, as in permanent translocation heterozygote *Rhoeo spathacea* (Golczyk et al. 2005). The rDNA distribution in tetraploid *Aconitum* species suggests the role of additional ribosomal sites in differentiation of chromosome pairs in these plants: in many instances, two chromosomes within a four-chromosome group showed the rDNA segment while two did not (Figs. 5 and 6).

The massive rDNA amplification in *A. firmum* and *A. plicatum* should result in a detectable increase of the genome size in both species, but Cx DNA values calculated for the analyzed species (5.71 and 5.61 pg in diploids; 5.40 and 5.31 pg in tetraploids) suggest instead a small reduction of the basal genome size in the tetraploids (Table 3). Genome downsizing is a well-known phenomenon detected in many polyploid plants (for review see Leitch and Bennett 2004), although in some plant genera, no discernible pattern in this direction has been observed (Grabowska-Joachimiak et al. 2006; Leitch et al. 2008; Klos et al. 2009; Anamthawat-Jónsson et al. 2010; Morozowska et al. 2015; Podwyszyńska et al. 2016). Most probably, global changes in genome size result from both the elimination and accumulation of different genomic sequences. Evidently, in tetraploid *Aconitum* species, the accumulation of rDNA repeats in new chromosomal sites was counterbalanced by the elimination of some other sequences from the genome. This may suggest the existence of mechanism that stabilizes the basal genome size in *Aconitum*. It is worth mentioning that the 1C_x_ value (5.44 pg) in *Aconitum vulparia* belonging to the subgen. *Lycoctonum* (the only *Aconitum* species analyzed so far in this respect: Siljak-Yakovlev et al. 2010) is located within the range of values given here (5.31–5.71 pg).

The gain or loss of different DNA sequences and reorganization of chromosomal loci in polyploids are parts of process called diploidization (Wolfe 2001). From a cytological point of view, it leads to the formation of well-differentiated chromosome pairs. This allows, among other advantages, to avoid pairing problems at meiosis and increase fertility. Although the diploidization subsequent to polyploidization plays an irrefutable role in releasing of evolutionary potential of newly formed polyploids, it still remains an under-studied topic in plants (Dodsworth et al. 2016).
